# The Pension Bureau and Dr. Spooner

**Published:** 1871-06

**Authors:** 


### The Pension Bureau and Dr. Spooner.

We copy from the *Commercial Advertiser* of June 16th, the following very sensible article, and call attention to it, since such an editorial article on medicine or medical policy is so rarely seen in the *Commercial Advertiser* or any other popular sheet. We do not introduce the article as covering the ground, but as a sign of returning consciousness in the management of a very influential political paper:—

WHERE WILL IT END?

"The physicians of the Homœopathic school are much elated over the restoration of Dr. Spooner, of Oneida. It will be remembered that this doctor was deprived of the office of Examining Surgeon for the Pension Bureau, under a decision of ex-Commissioner Van Aernam. The new Commissioner is more liberal in his treatment of the medical profession. With the approval of the Secretary of the Interior, the degraded Homœopathist has been restored to his position.

At first sight no good reason appears why the Government should require the medical men in its service to be of one school any more than it insists that the federal chaplains shall be of a certain religious sect. It looked like intolerance to close the doors of the Pension Bureau upon a regularly educated and competent physician, no matter what his method of practice might be.

It may now be regarded as decided that, in the department of the Interior at least, Allopaths and Homœopaths shall have equal chances.

But to what limit is this liberal treatment of different medical schools to go? If Allopaths and Homœopaths are admitted to office in the Pension Bureau, why not admit Hydropaths, Eclectics, Botanics, Electrics, and every other of the numerous schools into which the medical men have divided themselves? It may be an erroneous idea, and we hope that it is so; but it looks as though the apparent liberality of the new commissioner of Pensions would make him liable to be called upon to decide now amongst a score of disagreeing doctors. A movement in the direction of peace promises to give rise to any quantity of discord." * * * * * * * * * * *

Dr. Spooner, it is said, was not what is called a regularly educated physician at all, was not in fellowship with his own craft, and would have been dismissed on account of incompetency, from his own record. Comment upon this action in a medical journal is quite unnecessary. Physicians understand and instinctively pity.—Ed.

